# The profile of autistic traits in patients with psychotic disorders

**DOI:** 10.1192/j.eurpsy.2025.899

**Published:** 2025-08-26

**Authors:** M. Milovanovic, M. Vlaisavljevic, R. Grujicic, S. Perovic, V. Mandic Maravic

**Affiliations:** 1 Institute of Mental Health; 2 Psychiatry, School of Medicine, University of Belgrade, Belgrade, Serbia

## Abstract

**Introduction:**

Autistic traits are typical, but not pathognomonic for autism spectrum disorders (ASD) and they can also be observed in individuals with psychotic disorders (PD). The Adult Autism Spectrum Quotient (AQ) serves as a screening test for autism, assessing five categories of autistic traits (AT). Previous research has shown that both ASD and PD have significantly higher AQ scores compared to healthy population (1).

**Objectives:**

To evaluate the profile of AT in patients with PD compared to healthy controls (HC), as well as to compare the profiles of AT between individuals with schizophrenia and those with unspecified psychotic disorder.

**Methods:**

This cross-sectional study included 38 individuals with PD and 80 HC. The instruments used in the research included: AQ50, Social Adaptation Self-Evaluation Scale – SASS, and Sheehan Disability Scale.

**Results:**

Sociodemopraphics are shown in table 1. The PD group had significantly higher scores than the HC for the overall AQ score and its sub-scores, except for attention to detail (ATD) (Graph 1). In the whole sample, there was a significant negative correlation between AQ scores and social functioning (Pearson Correlation .331_,_ p=0,000). There were no differences between patients with schizophrenia and unspecified psychotic disorder regarding AQ score (p=0,466), while patients with schizophrenia showed significantly lower social and overall functioning (SASS total p=019; Sheehan total p=0,001).

**Table 1:** 

	**PD (n=38)**	**HC (n=80)**	**Test**	**P**
**Sex (male)**	22 (58,9%)	34 (41,4%)	X^2^=3.25	0,086
**Age (years)**	41,8 ± 12,8	34,3 ± 11,7	t=-3,22	0,06
**Treatment duration**	16,7 ± 9,9			
**ICD-10 diagnosis**				
*F20*	22 (56,4%)	-		
*F29*	17 (43,6%)	-		
**SASS total**	40,5 ± 7,4	44,7 ± 5,9	t=3,4	0,02
**Sheehan total**	13,3 ± 8,5

Graph 1 legend

AQSS – AQ social skills; AQAS – AQ attention switching; AQATD – AQ attention to detail; AQCOM – AQ communication; AQIMAG – AQ imagination

**Image 1:**

**Figure:**
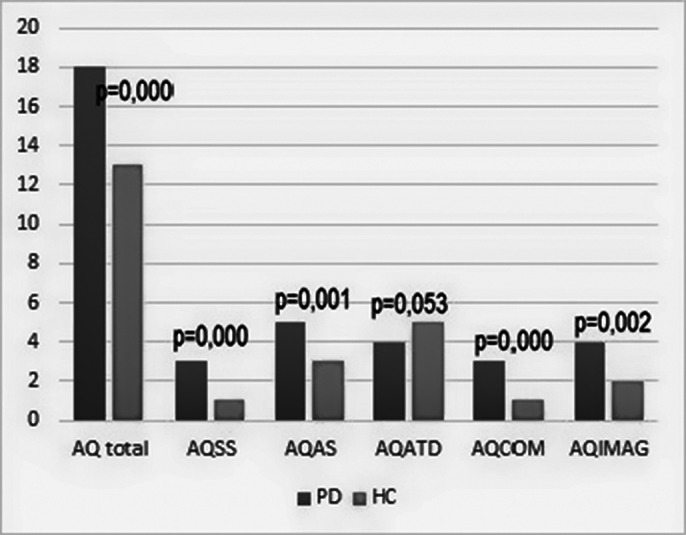

**Conclusions:**

Recognizing autistic symptoms in individuals with PD can be important for their social functioning, as well as for establishing an individualized approach to treatment, both pharmacological and non-pharmacological. It appears that AT impact social functionning differently in HC vs PD group. Further studies on correlation of AT with clinical outcomes in PD are warranted.

References

De Crescenzo F, Postorino V, Siracusano M, Riccioni A, Armando M, Curatolo P, Mazzone L. Autistic Symptoms in Schizophrenia Spectrum Disorders: A Systematic Review and Meta-Analysis. Front Psychiatry 2019;10:78.

**Disclosure of Interest:**

None Declared

